# Development and validation of a machine learning-based risk prediction model for cancer-related fatigue in ovarian cancer patients

**DOI:** 10.3389/fonc.2026.1815130

**Published:** 2026-06-04

**Authors:** Ru Feng, Zexuan Fan, Yuanyuan Pang, Qifan Ding, Qian Yue, Siqi Wei

**Affiliations:** School of Nursing, Lanzhou University, Lanzhou, Gansu, China

**Keywords:** cancer-related fatigue, machine learning, ovarian cancer, risk prediction model, support vector machine

## Abstract

**Background:**

Cancer-related fatigue (CRF) substantially compromises quality of life in ovarian cancer, yet reliable early detection tools remain inadequate. This study sought to develop a machine learning-based predictive model for CRF risk.

**Methods:**

We consecutively recruited 407 ovarian cancer patients from three tertiary hospitals in Lanzhou, China (October 2024–August 2025). Data were randomly partitioned into training (70%) and testing (30%) sets. Least Absolute Shrinkage and Selection Operator (LASSO) regression was applied for feature selection. Seven machine learning algorithms were developed, with the optimal model selected through comparative evaluation and subjected to SHAP interpretability analysis.

**Results:**

CRF prevalence was 39.6%. The Support Vector Machine (SVM) demonstrated superior overall predictive performance: AUC of 0.884, accuracy of 0.829, sensitivity of 0.816, specificity of 0.838, and F1 score of 0.792; good calibration (Brier score = 0.132); and decision curve analysis showed the highest net benefit across a wide range of threshold probabilities (0.05–0.85), indicating strong clinical utility. SHAP analysis identified serum calcium level, anxiety-depression status, red blood cell count, education level, cancer stage, medical payment method, and marital status as top predictive features.

**Conclusions:**

The SVM model exhibits robust predictive efficacy and good clinical utility, serving as a valuable tool for CRF risk stratification in ovarian cancer care. Early identification of high-risk patients enables targeted interventions to improve outcomes.

## 1 Introduction

Ovarian cancer is one of the most common malignant tumors of the female reproductive system. Its incidence rate ranks third after cervical cancer and endometrial cancer, while its mortality rate is the highest among gynecological malignancies (1, 2). In 2022, over 320,000 new cases of ovarian cancer were diagnosed worldwide, with a mortality rate as high as 63% (3). Due to its subtle early symptoms, insidious onset, and lack of effective screening methods, most patients are diagnosed at an advanced stage, with a five-year survival rate of only 46% (4).

Currently, ovarian cancer treatment primarily involves surgery combined with radiotherapy and chemotherapy, which has correspondingly extended patient survival. However, these therapeutic approaches are often accompanied by a range of side effects. Among these, cancer-related fatigue (CRF) has drawn widespread attention due to its persistent debilitating effects that severely impair patients’ quality of life (5). CRF is a persistent, debilitating, and subjective feeling of exhaustion or fatigue associated with cancer and its treatments. This fatigue is disproportionate to recent activity levels, occurs rapidly, is severe in intensity, persists over an extended period, and cannot be alleviated by rest (6). Research indicates that approximately 58% of ovarian cancer patients may experience CRF to varying degrees (7), not only does it exert long-term negative impacts on patients’ physical health, psychological state, and social functioning, significantly diminishing their quality of life, but it may even affect their life expectancy.

CRF is the result of the interaction between biological, psychological, and social factors. Physiologically, advanced age (8), late-stage cancer (9), anemia (10), and sleep disorders (11) are primary drivers. Psychosocially, patients with low education levels and low incomes struggle to effectively manage their disease due to resource constraints (12, 13), while married individuals face dual pressures of family support and reproductive health concerns (14). Furthermore, anxiety (15), depression (16), and fear of recurrence (17) form a vicious cycle with CRF, though psychological resilience and social support can mitigate these negative impacts as protective factors (18, 19). However, existing research predominantly focuses on single factors or traditional statistical models, making it challenging to capture the multidimensional and complex mechanisms underlying CRF development. Therefore, the development of a comprehensive, accurate, and convenient tool for predicting and assessing CRF in ovarian cancer patients is particularly urgent and necessary.

In recent years, risk prediction models have been extensively studied for forecasting the future progression of specific diseases or their complications. These models employ parametric, semiparametric, or nonparametric mathematical approaches to predict an individual’s current disease status or potential future outcomes (20), its core lies in integrating multiple predictive variables to construct models that estimate the incidence rate of specific clinical events, aiming to prevent and control the occurrence of diseases (21). Machine learning, as the fusion of computer science and statistics within the field of artificial intelligence, centers on training computer algorithms using data to recognize patterns and trends within that data, thereby generating predictive or decision-making models. Compared to traditional risk prediction models, machine learning-based predictive models demonstrate multiple advantages in clinical applications, such as large-scale data processing, modeling nonlinear relationships, personalized predictions, rapid model updates, and strong adaptability (22).

In summary, this study employed machine learning algorithms to construct a CRF prediction model for ovarian cancer patients. The algorithms included Logistic Regression (LR), Decision Tree (DT), Random Forest (RF), eXtreme Gradient Boosting (XGBoost), Light Gradient Boosting Machine (LightGBM), Support Vector Machine (SVM), and Artificial Neural Network (ANN). We evaluated the performance of different algorithms on specific datasets to identify the optimal model configuration for predictive efficacy. This approach effectively captures complex variables and potential associations related to CRF occurrence in ovarian cancer patients, providing robust data support for clinical decision-making. It advances personalized and precision management for ovarian cancer patients, ultimately improving their quality of life.

## 2 Materials and methods

### 2.1 Study design and participants

This cross-sectional study employed convenience sampling to select ovarian cancer patients treated at three tertiary general hospitals in Lanzhou City, Gansu Province, between October 2024 and August 2025, who met inclusion and exclusion criteria. Inclusion criteria were as follows: (a) Pathologically confirmed initial diagnosis of ovarian cancer; (b) Age ≥ 18 years; (c) Being conscious with basic communication and comprehension abilities; (d) Being aware of their actual diagnosis; (e) Providing informed consent and voluntarily participating in the study. Exclusion criteria were: (a) Having other major physical illnesses that prevent cooperation; (b) Having other malignant tumors; (c) Having a history of mental illness, psychiatric disorders, or cognitive impairment.

### 2.2 Sample size

The amount of data required for establishing a machine learning model is typically 10 to 20 times the number of included indicators. This study has 18 independent variables; calculating based on 20 times the number of indicators, a total of 360 cases are needed. Considering a 10% sample attrition rate, a total of 400 cases are required. This study ultimately included 407 patients.

### 2.3 Instruments

#### 2.3.1 General information questionnaire

Designed by the researchers themselves, the study included information such as age, educational attainment, marital status, monthly household income per capita, method of medical payment, tumor stage, disease duration, treatment modality, hemoglobin level, serum calcium, serum sodium, white blood cell count, serum albumin level, and serum creatinine.

#### 2.3.2 Cancer fatigue scale

This scale was specifically designed by Okuyama et al. (23) to assess fatigue symptoms in cancer patients. It employs a 4-point Likert scale and comprises three primary dimensions: physical fatigue, cognitive fatigue, and emotional fatigue, totaling 15 items. Higher scores indicate more severe fatigue. Based on the total CFS score, fatigue severity is categorized into four levels: scores below 5 indicate no fatigue, 6–15 indicate mild fatigue, 16–30 indicate moderate fatigue, and 30–60 indicate severe fatigue. The Chinese version of the CFS demonstrated test-retest reliability coefficients ranging from 0.55 to 0.77, showing good internal consistency across all dimensions and the total scale.

#### 2.3.3 Social support rating scale

Xiao Shuiyuan designed and introduced the Social Support Rating Scale in 1986 (24), which comprehensively assesses the level of support individuals receive in social environments. The scale comprises three dimensions: objective support, subjective perception of support, and utilization of social support resources, consisting of 10 items in total. A higher total score indicates a greater level of social support received by the individual. Based on the total score, social support can be categorized into three levels: low-level support (total score < 22), moderate-level support (23–44), and high-level support (45–66). The scale has undergone reliability and validity testing, demonstrating sound psychometric properties.

#### 2.3.4 Pittsburgh sleep quality index

The Pittsburgh Sleep Quality Index was developed by Dr. Buysse and his team at the University of Pittsburgh in 1989 to assess an individual’s sleep quality. This scale consists of 19 self-reported items and 5 observer-rated items, with 18 items grouped into 7 distinct factors. The sum of these factors constitutes the total scale score, ranging from 0 to 21 points. Higher scores indicate poorer sleep quality. Sleep quality is categorized into four levels based on the total score: 0–5 indicates good quality, 6–10 indicates fair quality, 11–15 indicates poor quality, and 16–21 indicates very poor quality. The PSQI demonstrates excellent reliability and validity, exhibits high internal consistency, and has been widely adopted in China.

#### 2.3.5 Hospital anxiety and depression scale

The scale was developed by Zigmond and Snaith (25) in 1983 to screen for anxiety and depression symptoms among non-psychiatric inpatients. It comprises two subscales: Anxiety and Depression, each containing seven items. Odd-numbered items assess anxiety levels, while even-numbered items assess depression levels, totaling 14 items. A 4-point Likert scale is used, with each item scored from 0 to 3 points. Both the anxiety and depression subscales range from 0 to 21 points. A score exceeding 8 points is considered indicative of positive anxiety or depression symptoms.

#### 2.3.6 Numeric rating scale

The Numerical Rating Scale is an internationally recognized pain assessment tool widely used in clinical practice (26). This scale quantifies patients’ pain intensity using numbers ranging from 0 to 10, where 0 represents “no pain” and 10 represents the “worst pain imaginable.” Based on NRS ratings, pain intensity is categorized into three levels: scores 0–3 indicate mild pain, 4–6 represent moderate pain, and 7–10 denote severe pain, where patients experience unbearable discomfort potentially accompanied by intense physiological and psychological reactions.

#### 2.3.7 Nutrition risk screening 2002

NRS 2002 is a nutritional screening tool proposed and recommended for use by the European Society for Parenteral and Enteral Nutrition (27) in 2002. NRS 2002 primarily assesses nutritional risk based on factors such as changes in patient weight, food intake, and disease severity. It is typically evaluated and completed by professionally trained healthcare personnel according to the patient’s actual condition. The NRS 2002 total score comprises three components: disease severity score, nutritional impairment score, and age score. A higher total score indicates greater nutritional risk. A total score ≥3 indicates nutritional risk, while a total score <3 suggests a lower current nutritional risk.

### 2.4 Data collection

This study employed face-to-face interviews for data collection. Prior to data collection, the research team conducted thorough discussions with the heads of relevant hospital departments to obtain departmental consent and support. During data collection, participants were selected strictly according to predetermined inclusion and exclusion criteria. Contact with patients was established with the assistance of nurses. Throughout the data collection process, the purpose and significance of the study were explained in detail to each participant, and strict confidentiality of personal information was assured. Informed consent was obtained from patients to safeguard their privacy rights. Participants completed questionnaires independently or with researcher assistance. To ensure consistency and accuracy, standardized instructions were provided before completion. Completed questionnaires were collected on-site, meticulously reviewed, and supplemented for any missing or omitted information. Each questionnaire was numbered for subsequent data entry and management.

### 2.5 Statistical analysis

#### 2.5.1 Descriptive analysis

Statistical analysis was performed using SPSS 25.0 software. Quantitative data were described as mean ± standard deviation, while categorical data were presented as frequency and percentage. Before risk factor analysis, data underwent K-S normality testing. Normally distributed data were analyzed using independent samples t-tests, while non-normally distributed data were assessed using the rank sum test.

#### 2.5.2 Data preprocessing

Using the missing value replacement principles from Python 3.10, discrete features with missing values are imputed using the mode, while continuous features are imputed using the mean. For outlier detection, the Isolation Forest algorithm is employed. Continuous features undergo data standardization, while discrete variables are processed using label encoding and one-hot transformation.

#### 2.5.3 Feature selection

Feature selection fundamentally involves identifying features relevant to the classification objective while eliminating irrelevant features and those with missing values exceeding 30%. LASSO regression is employed for feature screening to reduce overfitting and enhance model robustness, with variables exhibiting non-zero coefficients selected for model construction.

#### 2.5.4 Development and evaluation of predictive model

This study aims to predict the risk of CRF occurrence in ovarian cancer patients. Given the small sample size and the presence of multiple variables in this binary medical dataset, we applied several machine learning algorithms: Logistic Regression, Decision Tree, Random Forest, Extreme Gradient Boosting, Light Gradient Boosting Machine, Support Vector Machine, and Artificial Neural Network. Model evaluation metrics encompass discriminative power, calibration, and clinical utility. This study assesses models using Area Under the Curve (AUC), calibration curves, Decision Curve Analysis (DCA), Accuracy, Precision, Sensitivity, Specificity, and F1-score.

#### 2.5.5 Internal validation

The internal validation part of this study combined random splitting and cross-validation methods. The dataset was divided into training and testing sets at a ratio of 7:3. Five-fold cross-validation was used on the training set to evaluate model stability, with the average AUC as the performance metric. The final model was trained using the entire training set and evaluated on an independent testing set. All analyses were performed using Python 3.10.

#### 2.5.6 Interpretability analysis of the model

For the optimal predictive model identified through performance comparison, SHAP tools were employed to conduct interpretability analysis. This assessed the importance of each feature in the model’s decision-making process and provided a visual explanation of the role each feature plays in the model’s decision-making (28).

## 3 Results

This study included 432 ovarian cancer patients admitted for treatment at three tertiary-level Class A hospitals in Lanzhou City. Among them, 25 patients were excluded due to missing >30% of their case data. A total of 407 valid questionnaires were collected, yielding a valid response rate of 94.2%.

### 3.1 Participants’ characteristics

This study ultimately included 407 patients, among whom 161 developed CRF, yielding a CRF incidence rate of 39.6%. Detailed information on the participants is provided in **Table 1**.

**Table 1:** Basic characteristics of participants.

Variables	Total (n = 407)	Non CRF (n = 246)	CRF (n = 161)	Statistic	*P*
Marriage, n (%)				χ²=17.81	<.001
Unmarried	9 (2.21)	6 (2.44)	3 (1.86)		
Married	360 (88.45)	224 (91.06)	136 (84.47)		
Divorced	17 (4.18)	2 (0.81)	15 (9.32)		
Widowed	21 (5.16)	14 (5.69)	7 (4.35)		
Education, n (%)				χ²=27.64	<.001
Elementary school or lower	91 (22.36)	41 (16.67)	50 (31.06)		
Middle school	195 (47.91)	110 (44.72)	85 (52.80)		
High school	109 (26.78)	87 (35.37)	22 (13.66)		
University and above	12 (2.95)	8 (3.25)	4 (2.48)		
Average monthly household income per capita, RMB				χ²=3.08	0.379
< 1000	56 (13.76)	28 (11.38)	28 (17.39)		
1000–3000	246 (60.44)	154 (62.60)	92 (57.14)		
3001–5000	94 (23.10)	57 (23.17)	37 (22.98)		
> 5000	11 (2.70)	7 (2.85)	4 (2.48)		
Medical payment method				χ²=5.44	0.245
Self-payment insurance	31 (7.62)	24 (9.76)	7 (4.35)		
New rural cooperative medical system	276 (67.81)	165 (67.07)	111 (68.94)		
Urban residents’ basic medical insurance	68 (16.71)	39 (15.85)	29 (18.01)		
Urban employee’ basic medical insurance	22 (5.41)	11 (4.47)	11 (6.83)		
Commercial insurance	10 (2.46)	7 (2.85)	3 (1.86)		
Cancer stage				χ²=25.63	<.001
I	19 (4.67)	9 (3.66)	10 (6.21)		
II	73 (17.94)	62 (25.20)	11 (6.83)		
III	262 (64.37)	151 (61.38)	111 (68.94)		
IV	53 (13.02)	24 (9.76)	29 (18.01)		
Treatment method				χ²=6.65	0.036
Operation	47 (11.55)	25 (10.16)	22 (13.66)		
Chemotherapy	38 (9.34)	30 (12.20)	8 (4.97)		
Operation and Chemotherapy	322 (79.12)	191 (77.64)	131 (81.37)		
Social Support				χ²=36.47	<.001
Low	25 (6.14)	14 (5.69)	11 (6.83)		
Medium	150 (36.86)	63 (25.61)	87 (54.04)		
High	232 (57.00)	169 (68.70)	63 (39.13)		
Sleep quality				χ²=14.44	0.002
Good	39 (9.58)	22 (8.94)	17 (10.56)		
Medium	146 (35.87)	105 (42.68)	41 (25.47)		
Poor	121 (29.73)	60 (24.39)	61 (37.89)		
Very Poor	101 (24.82)	59 (23.98)	42 (26.09)		
Anxiety and Depression				χ²=61.85	<.001
No	274 (67.32)	202 (82.11)	72 (44.72)		
Yes	133 (32.68)	44 (17.89)	89 (55.28)		
Nutritional risk				χ²=6.92	0.009
No	246 (60.44)	136 (55.28)	110 (68.32)		
Yes	161 (39.56)	110 (44.72)	51 (31.68)		
Age	61.01 ± 7.64	60.09 ± 7.13	62.42 ± 8.20	t=-3.04	0.003
Disease duration	11.75 ± 8.14	11.06 ± 7.71	12.82 ± 8.67	t=-2.15	0.032
Hemoglobin	127.15 ± 10.07	128.43 ± 10.19	125.20 ± 9.60	t=3.20	0.001
Na	139.16 ± 3.04	139.26 ± 3.01	139.01 ± 3.09	t=0.82	0.415
White blood cell	6.76 ± 1.31	6.81 ± 1.25	6.70 ± 1.40	t=0.84	0.402
Albumin	39.88 ± 4.30	40.43 ± 4.31	39.04 ± 4.16	t=3.23	0.001
Cr	85.62 ± 6.69	84.51 ± 6.64	87.32 ± 6.42	t=-4.23	<.001
K	4.35 ± 0.39	4.36 ± 0.38	4.33 ± 0.42	t=0.75	0.452
Ca	2.36 ± 0.18	2.39 ± 0.17	2.32 ± 0.18	t=3.58	<.001
Platelet	212.19 ± 31.14	216.74 ± 29.97	205.24 ± 31.69	t=3.70	<.001
Red blood cell	4.26 ± 0.41	4.34 ± 0.41	4.15 ± 0.38	t=4.76	<.001
Pain	4.13 ± 1.96	3.64 ± 1.77	4.88 ± 2.00	t=-6.54	<.001

T, t-test; χ², Chi-square test; SD, standard deviation.

### 3.2 Feature variable selection

This study employed LASSO regression for feature selection and model optimization. The LASSO cross-validation curve indicates that seven features were ultimately selected: Anxiety and Depression, Cancer stage, Education, Serum calcium, Marital status, RBC, and Medical payment method. The weighted coefficients for each Lasso feature are presented in **Table 2**.

**Table 2:** Weighting coefficients for each feature of LASSO.

Variables	Weighting coefficients
Anxiety and Depression	0.403
Cancer stage	-0.348
Education	-0.307
Calcium	0.303
Marriage	0.273
RBC	-0.222
Medical payment method	0.156

**Figure 1:**
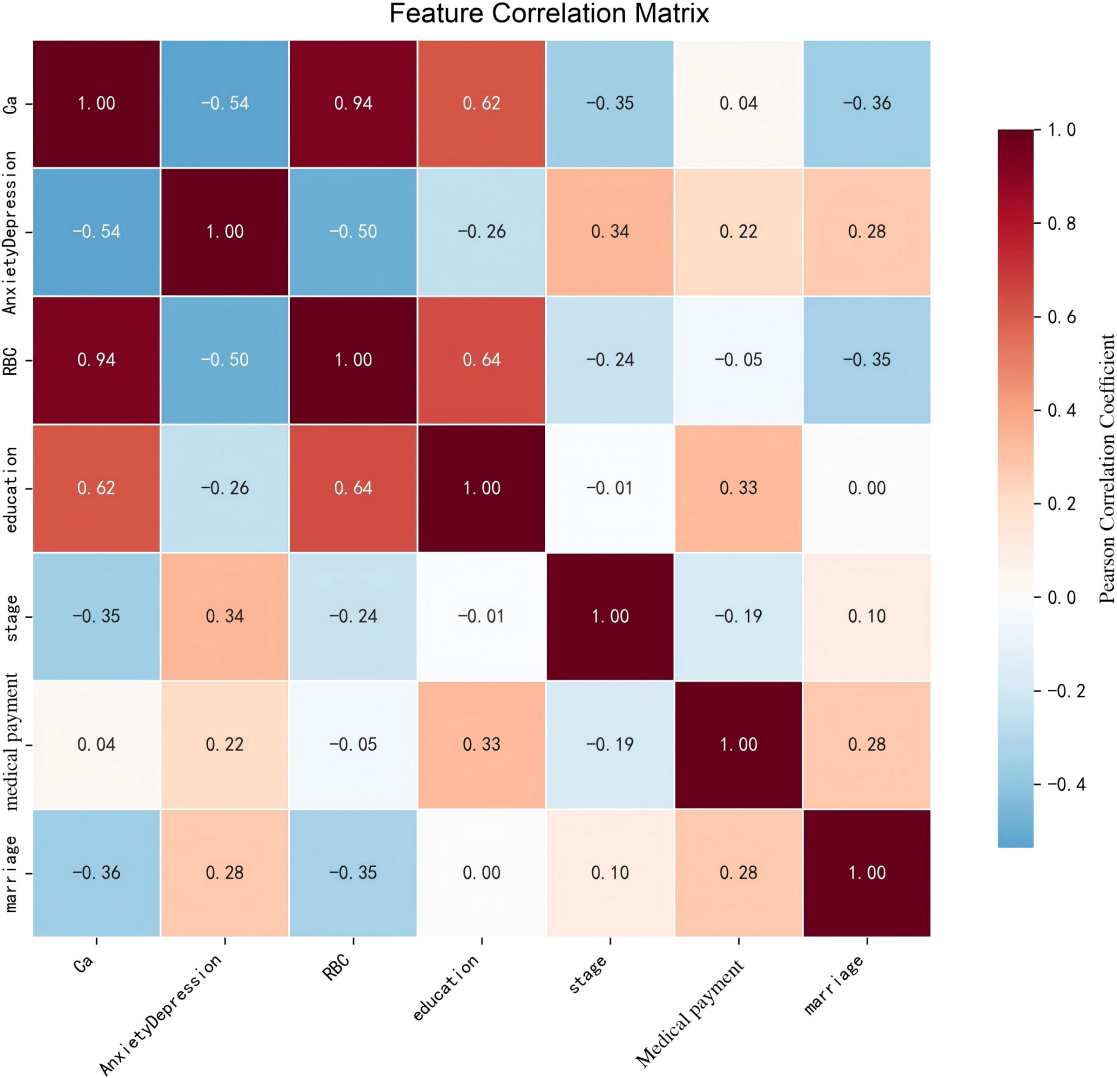
Feature correlation matrix of the seven LASSO-selected features.

To assess multicollinearity among the seven LASSO-selected features, we calculated the Pearson correlation matrix (**Figure 1**). The results showed a strong positive correlation between serum calcium (Ca) and red blood cell count (RBC) (r = 0.94), while all other pairwise correlations were below 0.6. Despite the high correlation between Ca and RBC, both features were retained by LASSO regularization, suggesting they may provide complementary predictive information reflecting distinct pathophysiological pathways. No significant multicollinearity issues were detected that would compromise model stability.

### 3.3 Development and evaluation of predictive model

Based on the predictive variables selected via LASSO regression, seven predictive models were constructed: LR, DT, RF, SVM, ANN, XGB, and LGBM.

#### 3.3.1 Hyperparameter tuning and cross-validation results

To identify the optimal hyperparameters for each model, we performed parameter search combined with five-fold cross-validation. As shown in **Table 3**, SVM achieved the highest mean AUC (0.873 ± 0.043) under five-fold cross-validation, followed by ANN (0.865 ± 0.046) and XGBoost (0.864 ± 0.067). Compared with default parameters, all models showed improved performance, with LightGBM demonstrating the most substantial improvement (+5.0%). SVM exhibited the smallest standard deviation (0.043), indicating the best stability.

**Table 3:** Hyperparameter tuning results and five-fold cross-validation performance for all models.

Model	Optimal CV AUC (mean ± SD)	Default CV AUC	AUC improvement	Key optimal hyperparameters
SVM	0.873 ± 0.043	0.855	+0.018 (+2.1%)	C=83.66, kernel=rbf, gamma=0.063
ANN	0.865 ± 0.046	0.855	+0.010 (+1.2%)	hidden_layer_sizes= (128), activation=relu, lr_init=0.012
XGBoost	0.864 ± 0.067	0.844	+0.020 (+2.4%)	n_estimators=292, max_depth=7, lr=0.087
LGBM	0.858 ± 0.066	0.817	+0.041 (+5.0%)	n_estimators=537, num_leaves=22, lr=0.237
RF	0.848 ± 0.051	0.821	+0.027 (+3.2%)	n_estimators=546, max_depth=5, min_samples_leaf=1
LR	0.842 ± 0.032	0.840	+0.001 (+0.2%)	C=0.495, penalty=l2, solver=lbfgs
DT	0.820 ± 0.063	0.790	+0.030 (+3.8%)	max_depth=9, min_samples_leaf=8, criterion=entropy

CV, cross-validation; SD, standard deviation; lr, learning_rate; improvement, (optimal - default)/default × 100%.

#### 3.3.2 ROC curve analysis

**Figure 2:**
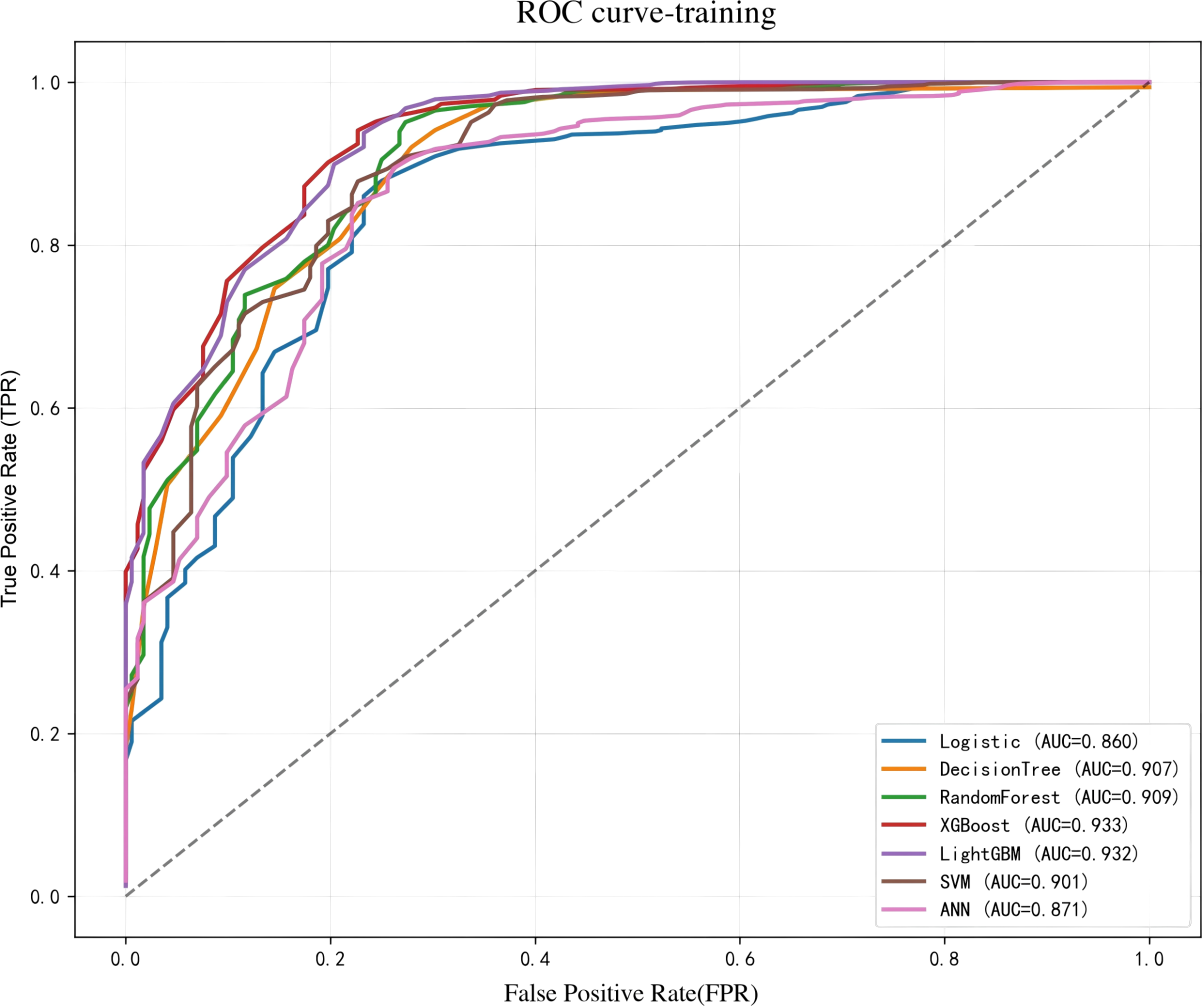
ROC curves of the seven prediction models on the training set.

**Figure 3:**
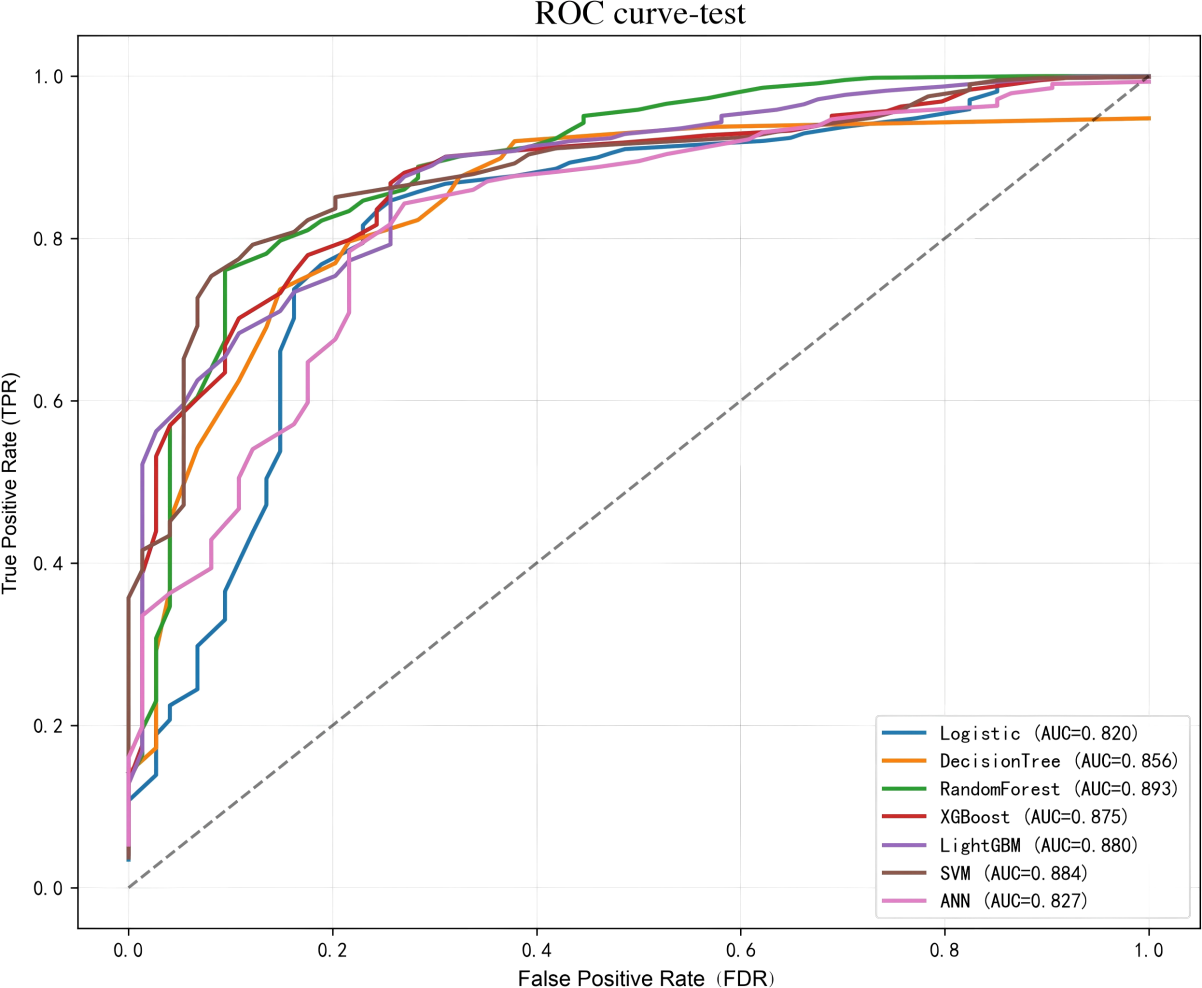
ROC curves of the seven prediction models on the test set.

On the training set (**Figure 2**), XGBoost achieved the highest AUC (0.933), followed by LightGBM (0.932), Random Forest (0.909), Decision Tree (0.907), SVM (0.901), ANN (0.871), and Logistic (0.860). On the test set (**Figure 3**), Random Forest ranked first (0.893), followed by SVM (0.884), LightGBM (0.880), XGBoost (0.875), Decision Tree (0.856), ANN (0.827), and Logistic (0.820). SVM showed the smallest training-to-test AUC decline (Δ=0.017), indicating the best generalization ability.

#### 3.3.3 Calibration curve analysis

**Figure 4:**
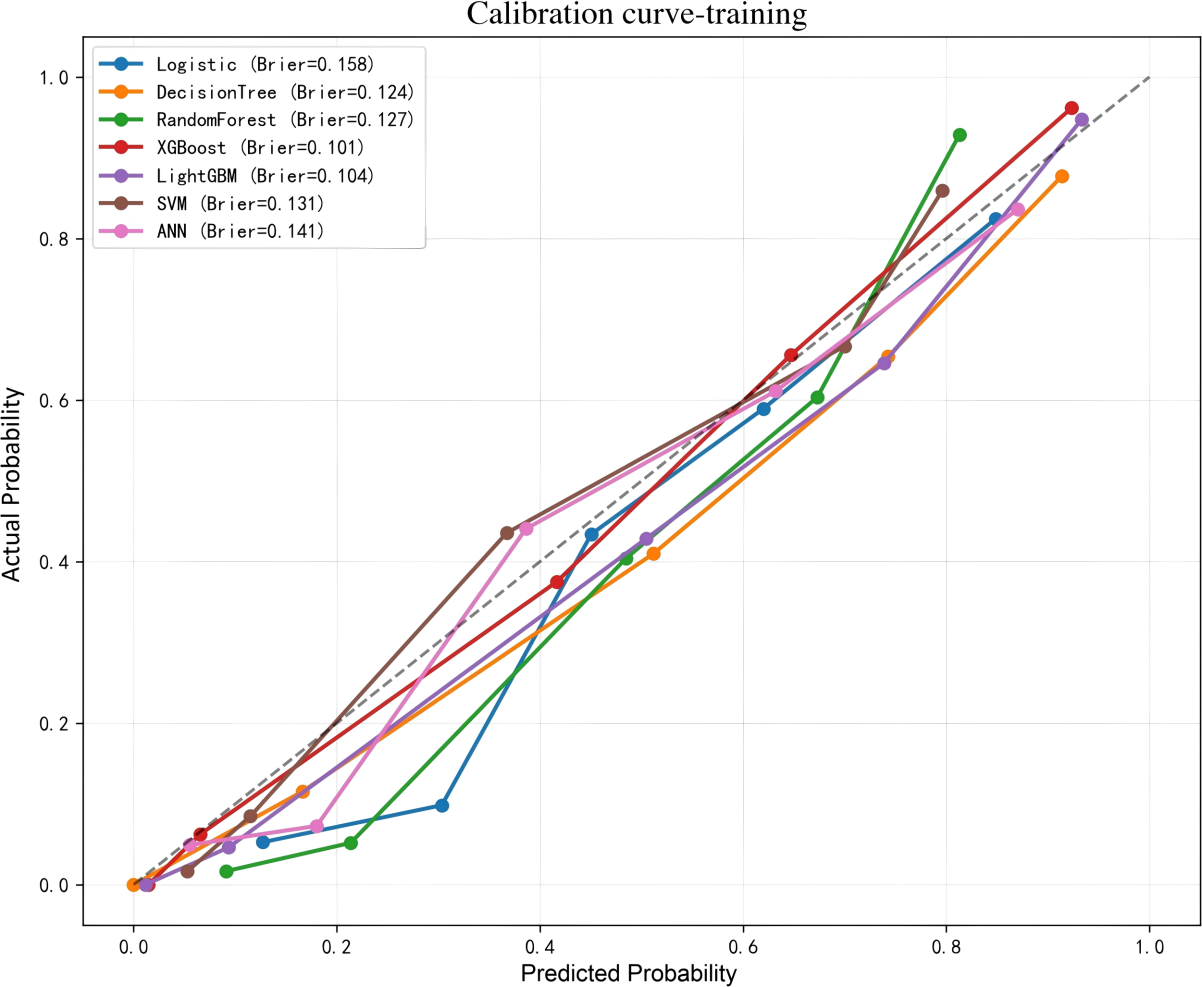
Calibration curves of the seven prediction models on the training set.

**Figure 5:**
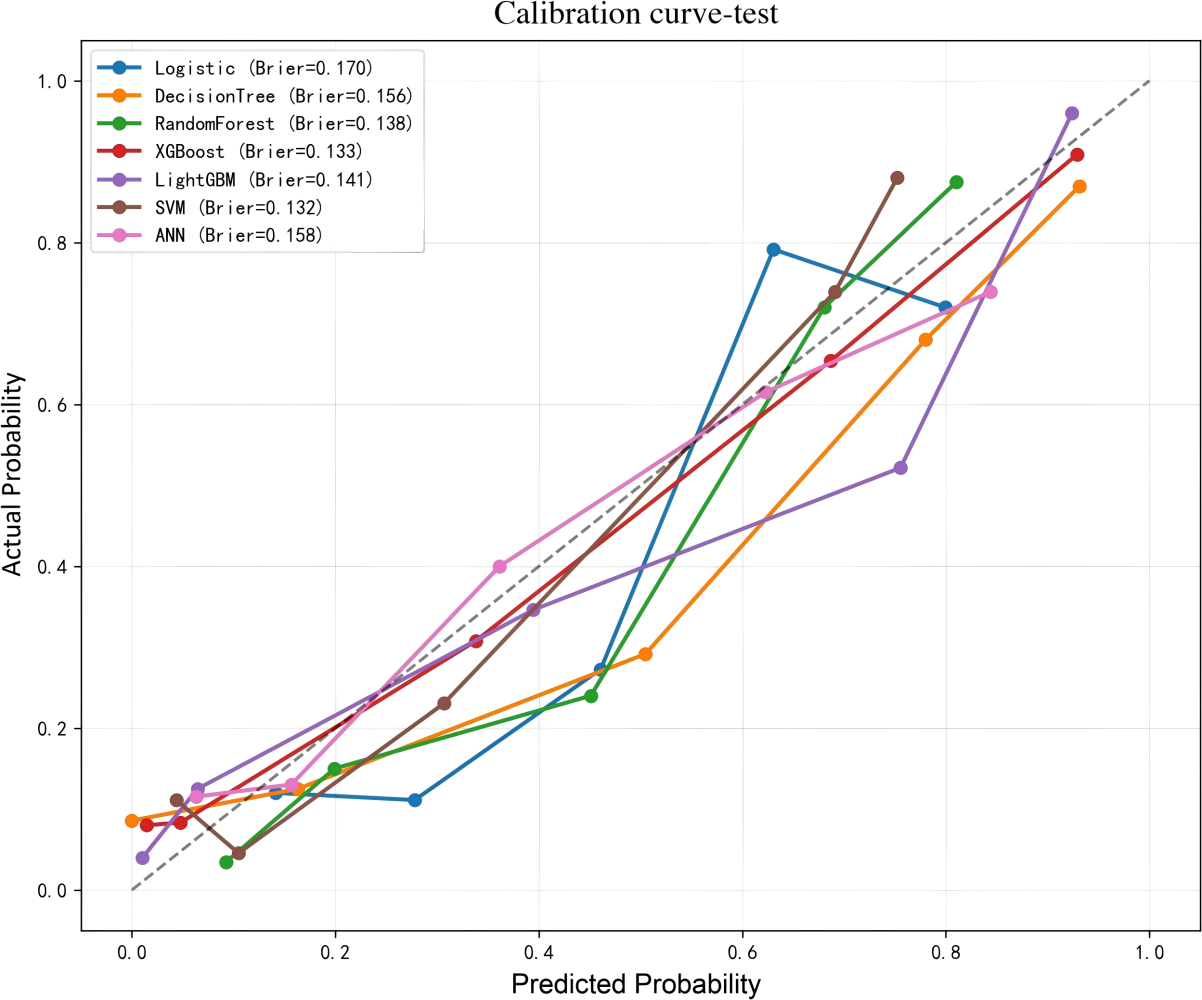
Calibration curves of the seven prediction models on the test set.

On the training set (**Figure 4**), XGBoost achieved the lowest Brier score (0.101), indicating the best calibration. On the test set (**Figure 5**), SVM achieved the lowest Brier score (0.132). SVM also showed the smallest training-to-test Brier difference (Δ = 0.001), demonstrating the most stable calibration. Although XGBoost was best calibrated on the training set, its performance notably degraded on the test set (Δ = 0.032), suggesting overfitting.

#### 3.3.4 Decision curve analysis

**Figure 6:**
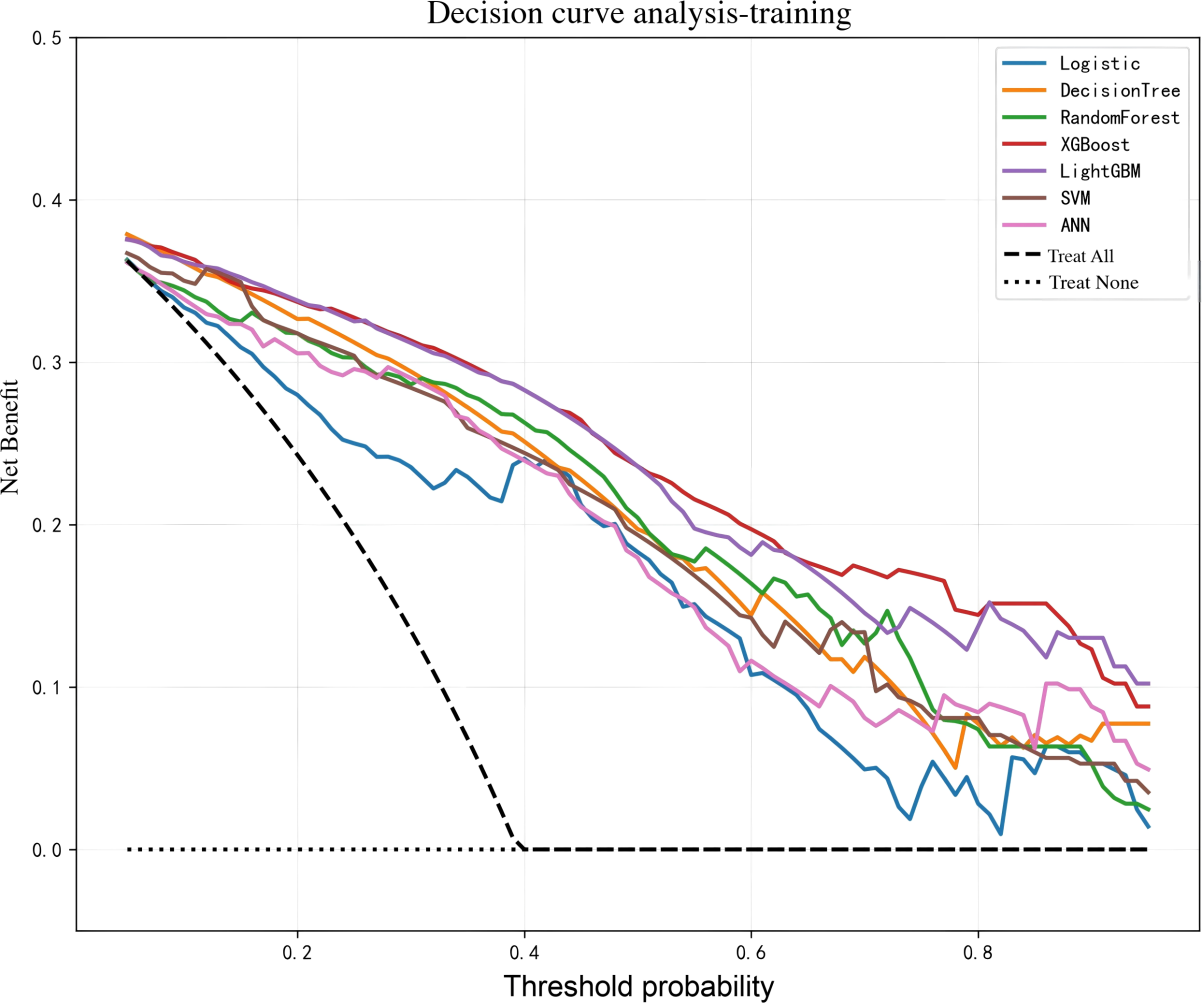
Decision curve analysis of the seven models on the training set.

**Figure 7:**
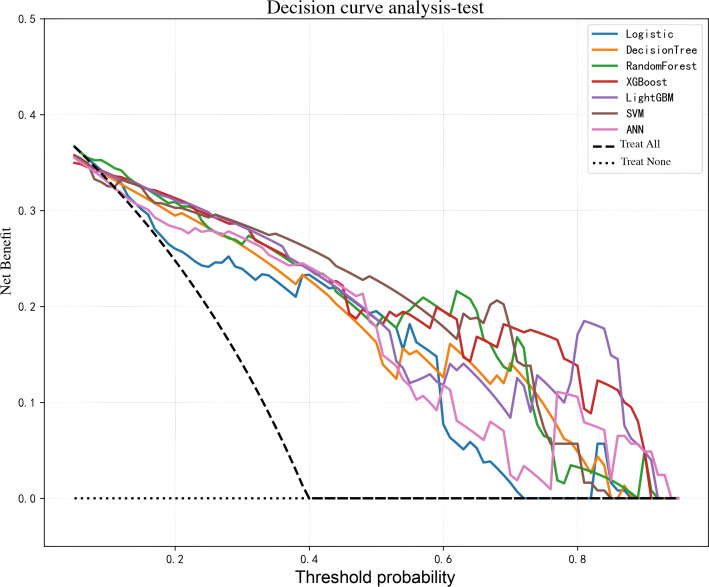
Decision curve analysis of the seven models on the test set.

Decision curve analysis (**Figures 6**, **7**) showed that SVM achieved the highest and most stable clinical net benefit on both training and test sets. On the test set, SVM yielded the highest net benefit across a wide range of threshold probabilities (0.05–0.85), substantially outperforming the “treat all” and “treat none” strategies. XGBoost and LightGBM performed well at low-to-moderate thresholds but showed lower net benefits at higher thresholds compared to SVM. Logistic regression and Decision Tree had limited clinical applicability, with narrower effective threshold ranges.

#### 3.3.5 Model performance evaluation

As shown in **Table 4**, on the training set, XGBoost achieved the highest AUC (0.933, 95% CI: 0.906–0.958), and LightGBM showed the highest sensitivity (0.955, 95% CI: 0.915–0.991). On the test set, Random Forest achieved the highest AUC (0.893, 95% CI: 0.828–0.946), while SVM ranked first in accuracy (0.829), precision (0.769), specificity (0.838), and F1 score (0.792).

**Table 4:** Predictive performance of 7 ML models in training and validation sets.

Set	Classifier	AUC (95% CI)	Accuracy (95% CI)	Precision (95% CI)	Sensitivity (95% CI)	Specificity (95% CI)	F1 (95% CI)
Training	LR	0.860 (0.816–0.904)	0.789 (0.743–0.835)	0.717 (0.632–0.797)	0.768 (0.691–0.845)	0.802 (0.742–0.859)	0.741 (0.678–0.800)
	DT	0.907 (0.875–0.939)	0.803 (0.754–0.852)	0.684 (0.609–0.759)	0.929 (0.879–0.973)	0.721 (0.655–0.788)	0.788 (0.730–0.839)
	RF	0.909 (0.876–0.940)	0.810 (0.768–0.852)	0.701 (0.627–0.773)	0.902 (0.847–0.955)	0.750 (0.686–0.817)	0.789 (0.733–0.839)
	XGB	0.933 (0.906–0.958)	0.842 (0.799–0.884)	0.764 (0.690–0.839)	0.866 (0.800–0.924)	0.826 (0.769–0.879)	0.812 (0.759–0.861)
	LGBM	0.932 (0.906–0.957)	0.842 (0.799–0.887)	0.728 (0.654–0.795)	0.955 (0.915–0.991)	0.767 (0.707–0.829)	0.826 (0.776–0.874)
	SVM	0.901 (0.864–0.934)	0.799 (0.754–0.845)	0.731 (0.647–0.807)	0.777 (0.696–0.852)	0.814 (0.757–0.869)	0.753 (0.688–0.812)
	ANN	0.871 (0.827–0.912)	0.785 (0.736–0.835)	0.718 (0.634–0.800)	0.750 (0.673–0.827)	0.808 (0.747–0.863)	0.734 (0.670–0.795)
Test	LR	0.821 (0.739–0.894)	0.797 (0.724–0.862)	0.707 (0.589–0.814)	0.837 (0.727–0.930)	0.770 (0.667–0.863)	0.766 (0.673–0.846)
	DT	0.856 (0.777–0.927)	0.764 (0.675–0.837)	0.652 (0.525–0.758)	0.878 (0.778–0.959)	0.689 (0.569–0.792)	0.748 (0.640–0.828)
	RF	0.893 (0.828–0.946)	0.780 (0.699–0.854)	0.677 (0.547–0.784)	0.857 (0.750–0.944)	0.730 (0.616–0.829)	0.757 (0.655–0.836)
	XGB	0.875 (0.802–0.938)	0.789 (0.707–0.862)	0.709 (0.579–0.827)	0.796 (0.679–0.904)	0.784 (0.679–0.871)	0.750 (0.647–0.836)
	LGBM	0.880 (0.806–0.939)	0.789 (0.707–0.862)	0.689 (0.554–0.796)	0.857 (0.756–0.947)	0.743 (0.634–0.843)	0.764 (0.659–0.843)
	SVM	0.884 (0.808–0.944)	0.829 (0.764–0.894)	0.769 (0.647–0.873)	0.816 (0.700–0.917)	0.838 (0.743–0.916)	0.792 (0.689–0.872)
	ANN	0.827 (0.747–0.900)	0.780 (0.699–0.854)	0.704 (0.582–0.814)	0.776 (0.653–0.894)	0.784 (0.686–0.875)	ff0.738 (0.633–0.826)

CI, confidence interval. Data are presented as point estimate (95% confidence interval). Confidence intervals were estimated using 1,000 bootstrap resampling iterations. LR, Logistic Regression; DT, Decision Tree; RF, Random Forest; XGB, XGBoost; LGBM, LightGBM; SVM, Support Vector Machine; ANN, Artificial Neural Network.

#### 3.3.6 Comprehensive evaluation and model selection

Seven models were systematically evaluated across multiple dimensions, including cross-validation stability, discriminative ability, calibration, clinical utility, and classification performance. In terms of cross-validation stability, SVM exhibited the smallest standard deviation (0.043) with fold-wise AUCs ranging from 0.806 to 0.915, indicating the best stability. Regarding discriminative ability, although Random Forest achieved a slightly higher test AUC (0.893 vs. 0.884), SVM showed the smallest AUC decline from training to test sets (Δ = 0.017), demonstrating superior generalization. For calibration, SVM achieved the lowest Brier score (0.132), indicating the best agreement between predicted probabilities and actual outcomes. In clinical utility, decision curve analysis showed that SVM achieved the highest net benefit across a wide range of threshold probabilities (0.05–0.85), demonstrating the strongest clinical applicability. Regarding classification performance, SVM ranked first on the test set in accuracy (0.829), precision (0.769), specificity (0.838), and F1 score (0.792). In summary, SVM demonstrated the most balanced and excellent performance across stability, generalization, calibration, clinical utility, and classification metrics. Therefore, SVM was selected as the optimal model for predicting cancer-related fatigue risk in ovarian cancer patients, and SHAP analysis was further performed for model interpretability.

### 3.4 Interpretation of the model

**Figure 8:**
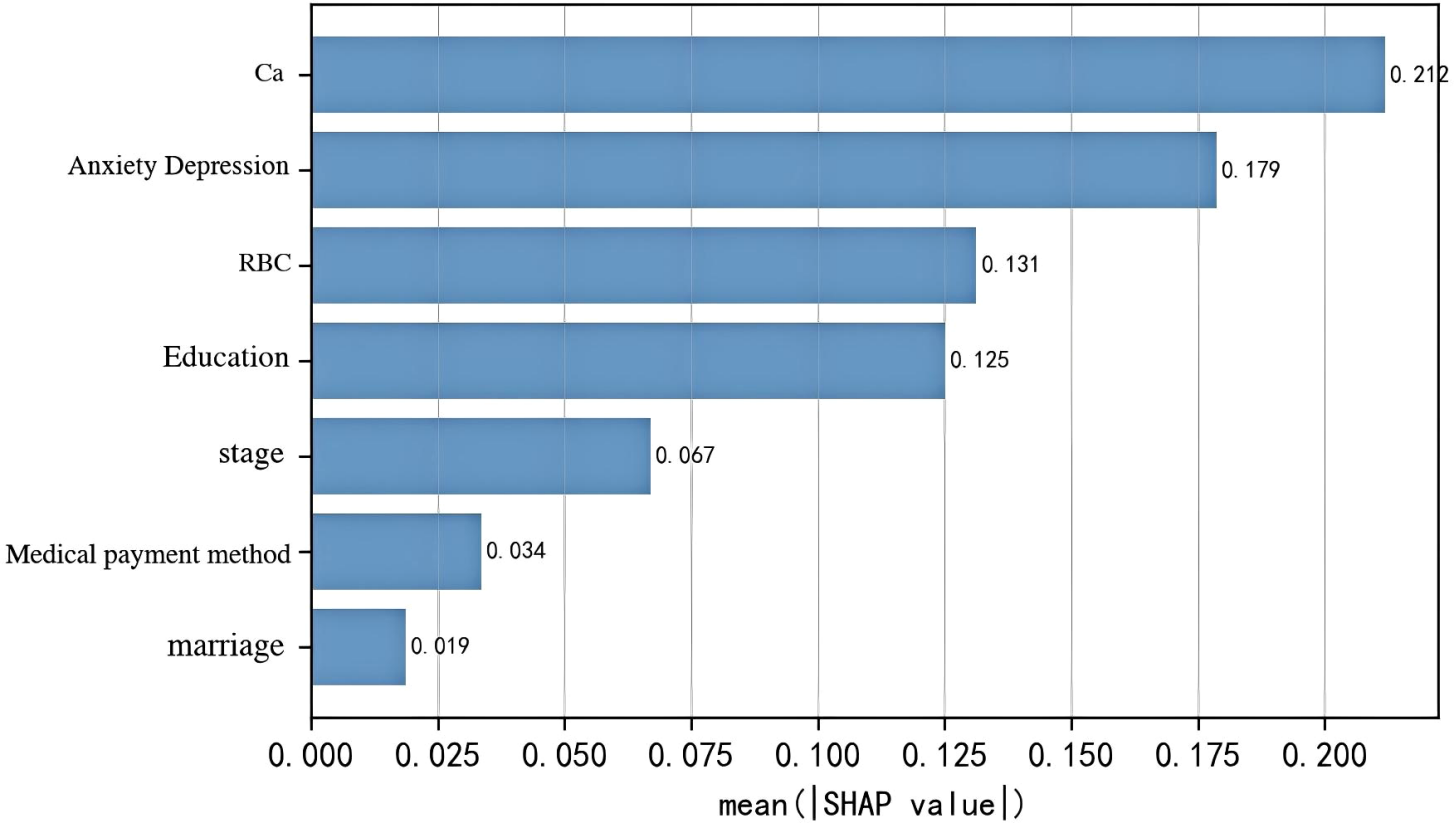
SHAP feature importance bar plot of the optimal SVM model.

**Figure 9:**
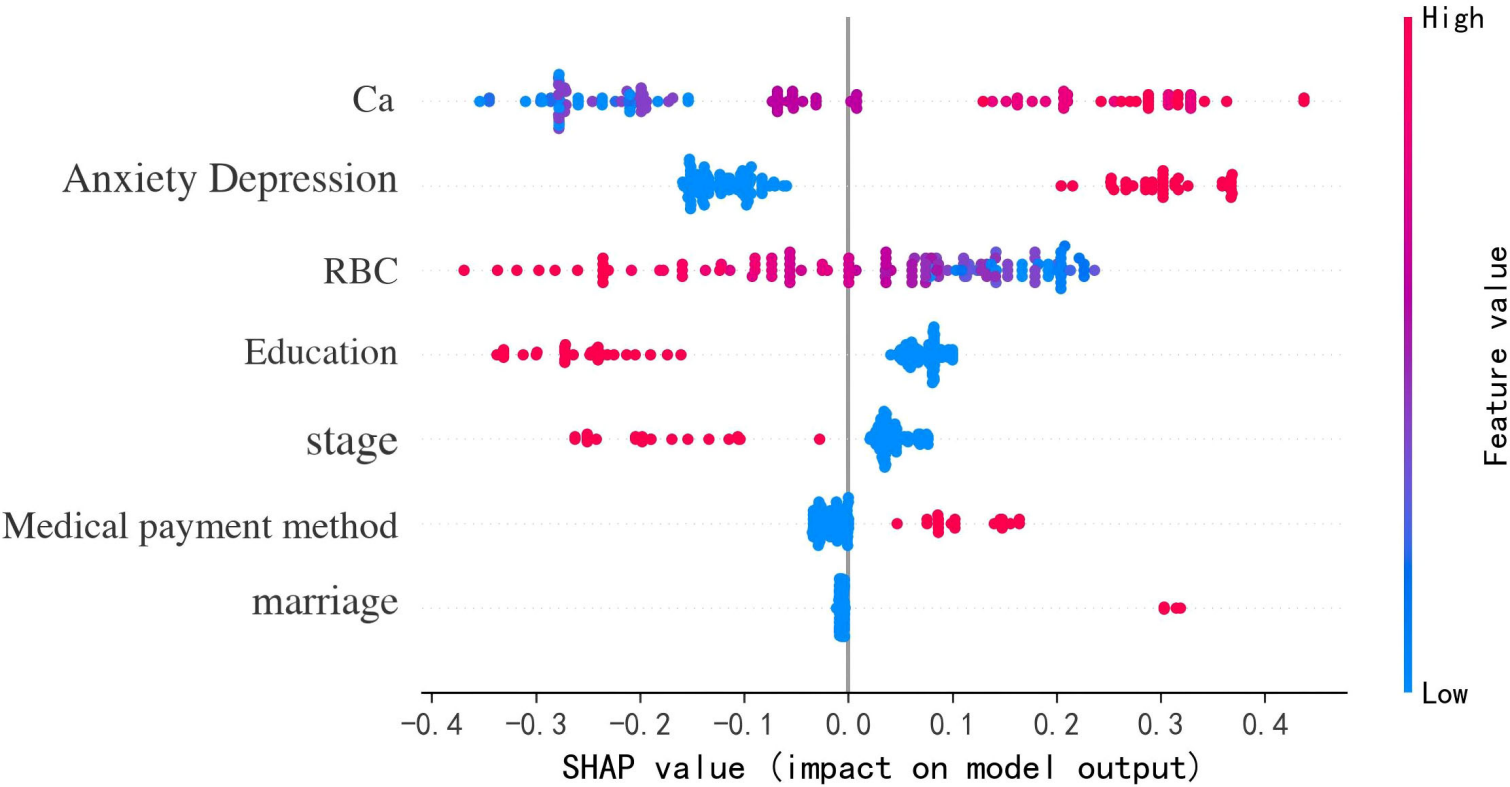
SHAP swarm plot of the optimal SVM model for CRF prediction.

SHAP analysis revealed that serum calcium (0.212), anxiety-depression (0.179), and RBC count (0.131) were the top three important predictive features in the optimal model (SVM) (**Figure 8**). The swarm plot (**Figure 9**) showed that high calcium, presence of anxiety-depression, and low RBC count increased CRF risk (SHAP values > 0), while higher education level and earlier cancer stage decreased risk (SHAP values < 0). Additionally, better medical payment method and married status had relatively small contributions to model predictions, both showing mild protective effects.

## 4 Discussion

This study preliminarily constructed a machine learning-based risk identification model for CRF in ovarian cancer patients. Feature selection was performed using LASSO regression, followed by model development with seven machine learning algorithms: LR, DT, RF, SVM, ANN, XGBoost, and LightGBM. Among these seven models, SVM demonstrated the best overall predictive performance. SHAP interpretability analysis of the SVM model, combined with LASSO regression results, identified seven key predictive factors: calcium level, anxiety and depression, red blood cell count, education level, cancer stage, medical payment method, and marriage.

### 4.1 Current status of CRF in ovarian cancer patients

The incidence of CRF among ovarian cancer patients in this study was 39.6%, lower than the findings reported by Hare.et.al (29).(54%). CRF is a subjective experience, and the discrepancy in findings may relate to assessment tools and patients’ states during evaluation. Additionally, compared to Western countries, Chinese patients, influenced by traditional beliefs, tend to suppress symptom expression to avoid adding to their family’s burden (30). Moreover, within China’s medical context characterized by “doctor authority,” patients may be inclined to provide “socially expected answers” when questioned (31).

### 4.2 The ovarian cancer CRF risk prediction model constructed based on SVM demonstrated the best performance

Among the seven models, SVM demonstrated the most comprehensive and optimal predictive performance. ROC curve analysis indicates that SVM achieved an AUC of 0.901 on the training set and 0.884 on the test set, demonstrating exceptional discrimination capability and sound generalization performance without significant overfitting. Calibration curves further revealed that SVM exhibited the lowest Brier score on the test set (0.132), indicating optimal consistency between predicted and actual observed probabilities and more reliable probability outputs. DCA analysis confirmed that across a broad range of threshold probabilities (approximately 0.05–0.85), the SVM’s net benefit curve outperformed other models, significantly surpassing the extreme strategy lines of “treat all” and “treat none” to deliver maximum net benefit for clinical decision-making. Regarding comprehensive performance metrics, SVM achieved the highest accuracy (0.829), precision (0.769), specificity (0.838), and F1 score (0.792) on the test set, ranking first among all models in these metrics. Given SVM’s comprehensive advantages across three dimensions—discriminative power, calibration, and clinical utility—its ability to effectively capture nonlinear interactions among the seven key features selected by LASSO and fully extract predictive information from limited features ultimately led to its selection as the optimal model for predicting CRF in ovarian cancer patients. SVM demonstrates promising clinical application prospects and significant value for broader implementation.

### 4.3 Predictors of complete response in ovarian cancer patients

The SVM model constructed in this study demonstrated significantly superior performance compared to traditional statistical methods for predicting CRF risk in ovarian cancer patients, exhibiting higher accuracy and stability. To enhance the model’s clinical credibility and practical value, the SHAP method was employed for in-depth analysis. SHAP interpretability analysis identified calcium ion levels, anxiety and depression, red blood cell, education, cancer stage, medical payment method, and marriage as key predictors of CRF in ovarian cancer patients, with average SHAP values of 0.212, 0.179, 0.131, 0.125, 0.067, 0.034, and 0.019, respectively.

#### 4.3.1 Calcium

In SHAP analysis, blood calcium ion levels emerged as the variable with the highest predictive value. When exceeding the normal range, higher calcium ion concentrations correlate with a greater probability of developing CRF. Hypercalcemia occurs in 2–30% of cancer patients and often indicates tumor progression or advanced disease. Fatigue symptoms can appear even with mild hypercalcemia (>10.5 mg/dL) and worsen as serum calcium levels rise (32). Ovarian clear cell carcinoma cells cause humoral hypercalcemia of malignancy through paracrine secretion of parathyroid hormone-related protein (PTHrP) (33). Hypercalcemia directly inhibits neuromuscular function. Excess extracellular calcium blocks voltage-gated sodium channels, disrupts transmembrane calcium transport, and impairs neuromuscular junction transmission, leading to muscle weakness, fatigue, and diminished reflexes (34). In addition, ovarian clear cell carcinoma frequently co-secretes granulocyte colony-stimulating factor (G-CSF), resulting in a dual paraneoplastic syndrome of hypercalcemia and leukocytosis, which significantly exacerbates cancer-related fatigue through increased systemic inflammatory burden (33). Furthermore, calcium homeostasis imbalance may contribute to CRF development through multiple pathways, including impaired muscle contraction, disrupted mitochondrial energy production, altered neuroendocrine regulation, and amplified inflammatory signaling (35).

#### 4.3.2 Depression and anxiety

Depression and anxiety are significant predictors of CRF. The prevalence of depressive symptoms among ovarian cancer patients in China reaches 47.0%, while anxiety symptoms reach 51.5% (36). As the core organ of female reproduction and sexual characteristics, the ovary’s pathological changes are implicitly associated with the loss of female integrity. Patients must confront not only the disease itself but also endure the dual pressures of gender identity crisis and social role disruption, which exacerbates anxiety and depression. Multiple studies (37, 38)have demonstrated a strong correlation between anxiety/depression and fatigue. Scholars have proposed three potential causal pathways for the persistent association between depression and CRF (39): fatigue inducing depression, depression leading to fatigue, or shared etiological factors. However, existing evidence remains insufficient to establish a definitive causal direction. Notably, although depression has been confirmed as a major precipitating factor for chronic fatigue syndrome(CFS) (35, 40), the pathophysiology of CRF may be distinct and cannot be simply modeled after CFS causation. In contrast, the association between anxiety and CRF has received less attention, with previous studies often treating anxiety as a comorbid variable to depression or reporting combined anxiety scores. However, a few studies have identified specific associations between trait anxiety and CRF, as well as the predictive role of baseline anxiety on subsequent fatigue, depression, or anxiety (41–43). Given the stable correlation between anxiety and CRF, future research should incorporate anxiety as an independent psychological variable in exploring CRF mechanisms, rather than merely viewing it as a subordinate component of depression.

#### 4.3.3 Red blood cell

Lower red blood cell counts increase the risk of CRF in ovarian cancer patients. Dose-dense chemotherapy regimens are the standard treatment for ovarian cancer, with a high incidence of grade 3/4 anemia reaching 56.1% (44). A decrease in total red blood cell volume directly reduces blood oxygen-carrying capacity, leading to insufficient tissue oxygen supply. This forces the body to rely on anaerobic metabolism, where lactic acid accumulation inhibits muscle contraction and induces intense fatigue (45). Additionally, to compensate for impaired oxygen transport, the cardiovascular system must operate under sustained high load. Prolonged cardiac work increases significantly exacerbates patients’ subjective fatigue (46). Reduced red blood cells markedly decrease maximal oxygen uptake (VO_2_max) and anaerobic threshold, causing routine activities to exceed metabolic thresholds. This accelerates lactic acid accumulation and triggers extreme fatigue, leading to a vicious cycle of “de-adaptation” characterized by reduced activity, muscle atrophy, and further decline in cardiopulmonary function (47).

#### 4.3.4 Education

The findings of this study suggest that patients with a high school education level have a lower risk of developing CRF compared to those with a junior high school education level or below and those with a high school education level or above. Sun et al. (13)found in a study of Chinese American breast cancer survivors that fatigue scores were highest among those with a junior high school education level or below, while those with a graduate degree or higher had the lowest fatigue scores. This may be attributed to relatively lower health literacy among those with lower educational attainment, resulting in limited knowledge about recognizing and managing cancer-related fatigue. Additionally, lower educational attainment often coexists with socioeconomic disadvantages such as lower income and occupational stress, leading to multiple psychosocial pressures and creating a vicious cycle of “low education-low resources-high fatigue.” However, other studies indicate that higher education levels may paradoxically correlate with more severe CRF (48). This may stem from higher-educated patients possessing greater knowledge, predominantly engaging in intellectual work, enduring greater stress and psychological burdens, and harboring higher treatment expectations and disease awareness—all factors that can exacerbate fatigue. The relationship between educational attainment and CRF may exhibit a U-shaped or inverted U-shaped pattern: individuals with extremely low educational attainment experience high fatigue due to low health literacy and resource scarcity; while highly educated individuals may experience elevated fatigue due to heightened disease awareness and psychological burden. Regardless, targeted fatigue education interventions effectively reduce fatigue intensity and distress (49), indicating that health literacy serves as a modifiable target for improving cancer-related fatigue. Clinicians should prioritize fatigue management education for patients with lower educational attainment.

#### 4.3.5 Cancer stage

Patients with stage II ovarian cancer have a lower risk of developing cancer-related fatigue compared to other stages. Existing studies indicate (9) that FIGO staging of ovarian cancer is positively correlated with cancer-related fatigue. FIGO stage > stage I is an independent predictor of receiving more fatigue-related management, suggesting that fatigue symptoms are more pronounced in advanced-stage patients. Advanced-stage patients exhibit significantly increased tumor burden, accompanied by substantial release of pro-inflammatory cytokines. This exacerbates fatigue by activating vagal afferent fibers and the central nervous system (50). Additionally, advanced-stage patients often undergo more extensive cytoreductive surgery and receive more cycles of chemotherapy (frequently >6 cycles), both of which can induce or worsen fatigue (51). Furthermore, HPA axis dysfunction is commonly observed at diagnosis in advanced ovarian cancer patients, manifested as flattened diurnal cortisol rhythms, which correlate significantly with fatigue severity (52). The higher CRF rates in stage I patients compared to stage II patients may stem from the sudden nature of diagnosis in stage I cases—often detected during routine check-ups or incidentally—leaving patients psychologically unprepared and experiencing intense acute stress responses (53). This leads to poor psychological adaptation and amplified fatigue perception. In contrast, stage II patients typically present with pelvic symptoms (e.g., abdominal distension, pain), allowing for some anticipation of disease prior to diagnosis and a relatively milder psychological impact. This suggests that clinical practice should prioritize psychological support for early-stage ovarian cancer patients, rather than solely focusing on physical symptoms in advanced-stage patients.

#### 4.3.6 Medical payment method and marriage

Medical payment method and marriage exert a relatively minor influence on the CRF model. Previous research (54) indicates that lack of health insurance is a significant risk factor for CRF. Uninsured patients experience heightened fatigue due to increased financial stress, which limits access to necessary medical services and leads to significantly elevated fatigue levels. Unmarried patients exhibit higher CRF levels. Married ovarian cancer patients demonstrate higher five-year survival rates than unmarried patients (55). Marriage provides crucial emotional support and practical care, whereas unmarried patients lack daily emotional support, experience heightened loneliness, and suffer increased emotional exhaustion (56).

## 5 Limitations

This study has several limitations. First, the sample size is relatively modest for machine learning applications, which may affect model stability and generalizability. Second, the cross-sectional design restricts causal inference regarding CRF. Given CRF’s dynamic evolution and potential bidirectional interactions with factors like anxiety and depression, future research should collect repeated measurements at multiple time points. Prospective cohort studies are needed to clarify temporal relationships and develop dynamic predictive models. Third, the sample originates from three hospitals in Northwest China, limiting regional representativeness. Additionally, some predictor variables are not routinely tested in healthcare settings globally, necessitating caution when extrapolating findings. Finally, the model’s generalizability across different racial and genetic backgrounds requires validation. Future studies should conduct external validation through multicenter, multinational, and multiregional research. Furthermore, exploring simplified models using more readily available variables could enhance clinical applicability.

## 6 Conclusion

The CRF risk prediction model for ovarian cancer patients constructed using the SVM algorithm demonstrated excellent predictive performance, achieving an AUC of 0.884, accuracy of 0.829, sensitivity of 0.816, specificity of 0.838, and an F1 score of 0.792 on the test set, with overall predictive capability outperforming other models. SHAP interpretability analysis identified serum calcium level, anxiety-depression status, red blood cell count, education level, cancer stage, medical payment method, and marital status as key predictive variables for CRF in ovarian cancer patients, with the combined contribution of calcium, anxiety-depression, and red blood cell count exceeding 60%. This model provides robust data support for clinical decision-making, advancing personalized and precision management of ovarian cancer patients to ultimately improve their quality of life.

## Data Availability

The raw data supporting the conclusions of this article will be made available by the authors, without undue reservation.
